# Flavor Profile Analysis of Instant and Traditional Lanzhou Beef Bouillons Using HS-SPME-GC/MS, Electronic Nose and Electronic Tongue

**DOI:** 10.3390/bioengineering9100582

**Published:** 2022-10-20

**Authors:** Zheqi Zhang, Jiaolong Jiang, Mingwu Zang, Kaihua Zhang, Dan Li, Xiaoman Li

**Affiliations:** 1 China Meat Research Center, Beijing 100068, China; 2 Gansu Longcuitang Nutrition and Health Food Co., Ltd., Lanzhou 730030, China

**Keywords:** Lanzhou beef bouillon, flavor profiles, HS-SPME-GC/MS, electronic nose, electronic tongue

## Abstract

The volatile profiles and taste properties of Lanzhou beef bouillons prepared with traditional (A1–A8) and modern (B1, B2) processing methods were evaluated. A total of 133 volatiles were identified: olefins, aldehydes and alcohols from spices in traditional bouillons were significantly higher (*p* < 0.05) than those in instant bouillons. The characteristic volatile substances in traditional beef bouillons were eucalyptol, linalool, 2-decanone, β-caryophyllene and geraniol; instant bouillons lacked 2-decanone and β-caryophyllene, and the contents of the other three substances were low. PCA (principal component analysis) and CA (clustering analysis) showed that the instant bouillons have a similar volatile profile to traditional bouillons, and the results of E-nose and sensory evaluation also supported this conclusion. The E-tongue showed that the taste profiles of instant bouillons were significantly different from those of traditional bouillons, mainly due to lack of umami; however, sensory evaluation revealed that taste differences were not perceptible.

## 1. Introduction

Lanzhou beef noodles, originated in Lanzhou city, Gansu Province, are one of the three most famous Chinese fast foods and have been consumed for more than 200 years [1]. After this long-term development, the cooking process and product quality have become highly standardized, and their processing technology has been listed as part of China’s intangible cultural heritage. Lanzhou beef noodles have a unique flavor because of the bouillon, which is different from regular beef broths. The most recent figures for 2019 show that in Lanzhou city alone, there are more than 1500 noodle restaurants, with one million bowls of beef noodles sold every day and an annual turnover of CNY 2 billion [2]. However, the current consumption of beef noodles is still mostly focused on the restaurant industry, and products, such as instant noodles, are lacking [3], which is mainly because the eating quality of previous products was not recognized by consumers. Although Lanzhou beef noodles have a relatively consistent eating quality, there are still subtle differences in the flavor profiles of products from different businesses due to minor differences in ingredients and processing processes. Therefore, the flavor quality of traditional beef noodles is also worthy of further study. Based on this current situation, instant food versions of Lanzhou beef noodles have been developed, and we analyzed the differences in flavor profiles between these versions and traditional beef noodles to investigate the effect of manufacturing on quality and provide a basis and direction for effectively improving product quality. Flavor profiles include aroma and taste; aroma can affect consumers’ first impression of food and purchase behavior, and taste can affect consumers’ sensory quality, overall acceptability and repurchase rates together with aroma [4,5].

Headspace solid-phase microextraction coupled with gas chromatography-mass spectrometry (HS-SPME-GC-MS) has been one of the most widely used techniques in recent years to identify volatile compounds in foods, such as meat, fish, fruits, tea, coffee and beer [6,7,8]. SPME is a promising sample preparation technique because it is simple to perform, avoids the use of organic solvents, has high sensitivity and selectivity, and is used for GC-MS preprocessing procedures [9]. GC-MS, with the advantages of high sensitivity and excellent selectivity, is one of the most efficient qualitative and quantitative methods for the analysis of volatile compounds. The E-nose and E-tongue, consisting of an array of sensors, signal acquisition systems and pattern recognition systems, were inspired by the way in which mammals recognize samples via olfaction and taste, respectively [5], and are largely utilized to detect volatiles and tastants. These devices can objectively evaluate the flavor profiles of foods and characterize the differences between them [10,11,12]. GC-MS combined with E-nose and E-tongue has been frequently used to characterize the flavor profiles of foods [13]. For example, Dong et al. [9] analyed the effect of the drying method on the volatile profiles and taste properties of roasted coffee beans. Yin, Lv, Wen, Wang, Chen and Kong [14] evaluated the volatile profiles and taste properties of Harbin red sausages. Zhang et al. [8] investigated the flavor profiles of fillets subjected to different drying methods, and all obtained promising results. This study aimed to characterize the flavor profile of traditional Lanzhou beef noodles and investigate the deficiencies of convenience products. Because the flavor of beef noodles mainly comes from boiling the beef bouillon by a special process and the flavor of the noodles is not prominent, the flavor characteristics of beef bouillons boiled by traditional and industrial processes were determined. Although there have long been studies on the flavor of broth [15,16,17,18], the flavor profile of this traditional food has not been studied. The information derived from this study will be helpful to clarify the flavor characteristics of Lanzhou beef noodles and provide a reference for the quality improvement of convenience products.

## 2. Materials and Methods

### 2.1. Materials and Chemicals

Beef bouillons boiled by traditional processes were purchased from eight different noodle shops in Lanzhou and were assigned as A1–A8. These eight stores were selected because they have high popularity and recognition in Lanzhou and may comprehensively reflect the flavor and quality of beef soup. Instant bouillons were assigned as B1–B2, and the cooking process and ingredients were basically the same as those of traditional beef bouillons. However, instant bouillons are boiled using a jacketed kettle, which can perform automatic timing and mixing, in contrast to a traditional saucepan. Then, the bouillons are concentrated to 1/3 of the original volume fraction and filled into the packaging bag under sterile conditions. Furthermore, instant bouillons are all sterilized at 120 °C for 15 min in a bath sterilizing pot (Zhucheng Shenlong Machinery Factory, Shandong, China). In addition, in the B2 group, concentrated beef bone broth was used instead of beef meat and bone broth.

The beef bouillon (purified water was added to the instant bouillon to restore it to its original concentration) was strained through gauze to remove solid residues and then cooled to 4 °C in a cold-water bath. After cooling, the bouillon was centrifuged at 2000× *g* for 10 min, and the supernatant was used for subsequent analysis.

2-Methyl-3-heptanone (99.0%) diluted in n-hexane, which was used as an internal standard (IS) and n-alkanes (C8–C20) (99.0%), which were used for retention indices, were purchased from Beijing Chemical Reagents Co., Ltd. (Beijing, China).

### 2.2. Analysis of Volatile Compounds

Volatile flavor compounds were extracted according to a previous method [8]. Processed beef noodle bouillon (15.00 g) was placed in 40 mL headspace sample vials (Supelco Inc., Bellefonte, PA, USA), 1 μL of 0.816 μg/μL 2-methyl-3-heptanone was added as an internal standard [19], and the vials were capped with a PTFE/silicone septum. After equilibration in a 45 °C water bath for 30 min, a SPME fiber (Supelco Inc., Bellefonte, PA, USA) coated with 50/35 μm carboxen/polydimethylsiloxane/divinylbenzene (CAR/PDMS/DVB) (2 cm) was placed in the headspace of the SPME vial for extraction for 30 min [20]. After extraction, the PDMS/DVB/CAR fiber was immediately inserted into the GC inlet for desorption at 230 °C for 5 min. The SPME needle was then removed and inserted into the GC inlet for desorption for 5 min.

Volatile compounds were analyzed using gas chromatography/mass spectrometry (GC-MS) (Thermo Fisher Scientific, Waltham, MA, USA) with a TG-Wax MS polar column (30 m × 0.25 mm, 0.25 μm, Thermo Fisher Scientific, Waltham, MA, USA); the carrier gas was high-purity helium (purity > 99.99%, Air Liquide Co., Ltd., Beijing, China) at a flow rate of 1.0 mL/min in splitless mode. The heating procedure included an inlet temperature of 230 °C, an initial column temperature of 40 °C, a holding time of 3 min, a temperature increase to 200 °C at a rate of 5 °C/min with a holding time of 1 min and a final temperature increase to 230 °C at a rate of 8 °C/min with a holding time of 3 min. The ion source temperature was 230 °C; other MS conditions included an electron energy of 70 eV, mass scanning range of 40–500 amu, and full-scan mode.

### 2.3. Identification of Volatile Compounds and Data Analysis

The volatile compounds were identified according to the method of Yin, Lv, Wen, Wang, Chen, and Kong [14], Wen, Hu, Zhang, Wang, Chen, and Kong [21] and Dong et al. [9] by comparing the experimental mass spectra with the mass spectra library of NIST17/Wiley (New York, NY, USA, 320k compounds, version 6.0) and/or via calculation of the retention index relative to a series of standard alkanes (C8–C20) to calculate the linear retention index (LRI). The results with a similarity degree >90% were retained. The volatile compounds were semi quantified by dividing the peak areas of the compounds of interest by the peak area of the IS and multiplying this ratio by the initial concentration of the IS (expressed as μg/kg) [14].

Odor activity values (OAVs) can represent the contribution of the volatile compound to flavor. OAVs were obtained by dividing the content of a volatile compound by its olfactory threshold, and the odor threshold values of these compounds were obtained from the relevant references. Compounds with an OAV >1 indicated that it may play an important role in the overall flavor [22].

### 2.4. Electronic Nose Analysis

A PEN3.5 electronic nose (Airsense Analytics GmbH, Schwerin, Germany) equipped with 10 metal oxide semiconductor (MOS) sensors, including W1C, W1S, W1W, W2S, W2W, W3C, W3S, W5C, W5S and W6S, for the recognition of volatile compounds was used for the E-nose analysis, according to the method of Wu, Yue, Xu, Zhang [23]. The sensors are described in Table 1. Processed beef noodle bouillon (1 g) was placed in a 5 mL airtight vial and incubated for 2 min at 50 °C. Then, a hollow needle with tubing was used to pierce the seal of the vial and absorb the volatile gases from the headspace at a constant rate. At the same time, clean air was supplied through a second hollow needle with a charcoal filter to replace the volatile gases. The measurement time was 90 s, and clean air was applied to flush the chamber to ensure that the sensor signals returned to baseline. The sensor information is detailed in Table 1. Five parallel samples were carried out for each group.

### 2.5. Electronic Tongue Analysis

The processed beef noodle bouillon (50.00 g) was measured using a TS-5000Z taste sensing system (Insent Inc., Atsugi, Japan). This E-tongue was equipped with five chemical sensors for umami, astringency, saltiness, sourness and bitterness and two reference electrodes. E-tongue analysis was performed according to a previous method [8]. Prior to analysis, the sensors were calibrated and tested to ensure that they were operating in the correct mV range. Between each measurement, the sensors were washed in a cleaning solution (90 s) and reference solutions (120 s + 120 s). The acquisition time was 90 s. Three parallel samples were analyzed in each group.

### 2.6. Sensory Evaluation

Preference testing was performed using 50 randomly selected indigenes from Lanzhou. The bouillons were evaluated for overall acceptability on a 9-point hedonic scale, where 1 = extreme dislike, 5 = neither like nor dislike, and 9 = extreme like. Sensory evaluation was performed according to the method of Hu et al. [24]. Ten qualified panelists (5 males and 5 females) received intensive training before sensory evaluation. The training was held for 1 week, 5 times, 1 h per session [25]. Odors and corresponding references are listed in Table 2. The sensory evaluation was based on a 10-point intensity scale from 1 (no intensity) to 10 (high intensity). Five-gram samples were randomly added to 10 mL clean, odorless glass bottles marked with randomized three-digit numbers. There were five-minute time intervals, and the panelists cleansed their mouths/taste buds with water. A professional sensory evaluation laboratory was used for training and sensory evaluation. All panelists signed written informed consent via the statement “I am aware that my responses are confidential, and I agree to participate in this survey” before sensory evaluation. The products tested were safe for consumption.

### 2.7. Statistical Analysis

All necessary data are expressed as the means ± standard deviation (SD). One-way analysis of variance (ANOVA) with Duncan’s multiple range tests were used for evaluating the differences between means (SPSS 19.0 SPSS Inc., Chicago, IL, USA). Differences were considered significant at *p* < 0.05. Principal component analysis (PCA) and clustering analysis (CA) were conducted by using MSpectrum Pattern software (PERSEE, Beijing, China). The data underwent normalization before analysis.

## 3. Results and Discussion

### 3.1. Volatile Compound Analysis

#### 3.1.1. Volatile Substance Contents

A total of 133 compounds, including 50 alkenes, 20 aldehydes, 18 alcohols, 10 esters, 7 ketones, 8 phenols, 8 ethers, 6 heterocycles, 4 aromatics, 1 sulfur-containing compound and 1 alkane, were detected in the 10 groups of beef bouillons. Detailed information is listed in Table 3. A total of 25 volatiles were detected in all samples. The contents of volatile compounds in the 10 groups were 3014.66 μg/kg, 6964.08 μg/kg, 1335.85 μg/kg, 1126.97 μg/kg, 2572.14 μg/kg, 2662.65 μg/kg, 1759.03 μg/kg, 2344.11 μg/kg, 367.55 μg/kg, and 1140.93 μg/kg. The content of volatile compounds in instant bouillons was lower than that in traditional bouillons in all 10 groups; in addition, the volatile compound contents in B1 were significantly lower than those in the other groups (*p* < 0.05), but there was no significant difference among B2, A3 and A4 (*p* > 0.05). A2 had significantly higher contents than the other groups (*p* < 0.05). It is possible that concentration and sterilization led to the loss of volatile substances in B1. Due to the use of concentrated bone broth, B2 may have had a higher volatile content compared with B1 before concentration and sterilization.

##### Alkenes

In all 10 groups, alkenes were the most abundant volatile compounds, and these substances included a large number of terpenes. Terpenes are widely distributed in plants, especially in spices, and studies have shown that their content is positively correlated with the weight of spices added to foods [26]. To remove the fishy smell of meat and provide a better aroma, a variety of spices are added to beef bouillons during boiling, increasing the olefin content. β-Pinene, 3-carene, α-phellandrene, limonene, styrene, p-menth-3-ene, copaene, β-elemene, β-caryophyllene, humulene, zingiberene, (Z, E)-α-farnesene, sesquiphellandrene, and α-curcumene were detected in all groups. The contents of these substances were lower in the instant bouillons than in the traditional bouillons, except for styrene in B2 group; in particular, the contents of copaene, β-caryophyllene, and humulene were significantly lower than those in most of the traditional bouillons. This difference may be due to the concentration process used in the processing of instant bouillons, as these substances evaporate with water.

##### Aldehydes

The aldehydes detected in the beef bouillons can be divided into two categories: some are produced by fatty acid oxidation and thermal degradation, such as octanal, 1-nonanal, and (E)-2-octenal, which are usually considered to be the major flavor contributors in meat products [27,28], and others, such as (E)-citral, (Z)-citral, and cinnamaldehyde, are from spices [29,30]. According to the contents of volatile substances that were detected in the two sources, the aldehydes in the beef bouillons mainly came from spices. Benzaldehyde, 2-propenal and cinnamaldehyde were detected in all 10 groups. Benzaldehyde, with aromas such as fat, nuts, and caramel, is an important aroma component in meat products and mainly comes from the Strecker degradation of phenylalanine [31]. The content in group B2 was significantly higher than that in the other groups, which may be due to the use of concentrated beef bone broth. Cinnamaldehyde is widely found in spices, and the odor is described as cinnamon or paint; this compound was also the main aldehyde in each group. 2-Propenal may be a derivative of benzaldehyde, but it has also been found in spices [28,31].

##### Alcohols

The current literature shows that alcohols can be divided into linear alcohols, which are mainly from lipid oxidation, and branched alcohols, which are the degradation products from the corresponding branched aldehydes [32,33]. The substances detected in each group included eucalyptol, linalool, terpinen-4-ol, α-terpineol, and geraniol, which are common volatile compounds in spices [28]. As shown in Table 2, the contents of the above substances in group B1 were lower than those in the other groups, and Group B2 had moderate to low levels. Eucalyptol, linalool, and geraniol have low olfactory thresholds, and their effect on aroma was obvious. Linalool is associated with waxy, green, aldehyde, berry and fresh woody fragrances, eucalyptol has a mint aroma, and geraniol has a sweet smell; these substances usually exist in spices, such as pepper, ginger and laurel [34]. The low content of these substances in instant bouillons may be related to the use of dehydrated coriander and garlic cloves, which may be lost during dehydration.

##### Esters, Phenols and Ketones

Esters are derived from the esterification of carboxylic acids and alcohols, and their contents were low in the B1 and B2 groups, most likely due to the low content of alcohols. Esters have sweet and fruity aromas, but because of their high smell threshold and low content, they have little effect on the flavor of the product. Although eight phenolic compounds were identified, similar to esters, they had little contribution to flavor, except for eugenol in the A5 and A6 groups. The contents of phenols in the two industrially produced beef bouillons were lower than those in traditional bouillons.

Regarding ketones, which are responsible for the fatty aroma in cooked meat, seven were detected, and 6-methyl-5-hepten-2-one was detected in all groups; however, the contents were low (maximum 12.68 μg/kg). According to Pham et al. [35], methyl ketones are related to the degree of lipid oxidation, and they may be generated by amino acid decomposition or the β-oxidation of fatty acids derived from triglycerides by heat treatment. In addition, a small amount of ketones come from spices, such as piperitone and camphor [36].

##### Heterocycles, Aromatics, Sulfides and Alkanes

Heterocyclic compounds are generally considered to be produced by the Maillard reaction of amino acids and reducing sugars, which can provide unique meat flavors [31]. Linalyl oxide comes from the oxidation of linalool in bouillons. Furan, an indicator of lipid oxidation in meat products, was only detected in the instant bouillons, meaning that the degree of lipid oxidation in instant bouillons is high [37], which may be due to heat sterilization. O-Cymene was detected in each group and was also the only heterocyclic compound in group B1. Sulfide is the most common dimethyl disulfide in meat products and was only detected in groups A1 and A3. One alkane was also detected; alkanes mainly come from the homolysis of alkoxy groups of fatty acids [31]. However, the olfactory threshold of alkanes is high, which is why they had little contribution to flavor. In general, the above substances had little effect on the flavor of the beef soup.

#### 3.1.2. Odor Activity Value Analysis

The odor perception of food is determined by both the contents and threshold values of volatile compounds [38]. OAV is the most commonly used index to evaluate the contribution of volatile compounds by combining these two parameters. Normally, OAV > 1 means that the compound contributes to the overall odor, and the contribution is positively correlated with OAV. All 78 volatile compounds with an OAV > 0.01 are listed in Table 4. There were 30 key volatile compounds with OAVs > 1, but most of them had higher OAVs in only one or several groups. In accordance with a previous study, dimethyl disulfide, β-myrcene, limonene, eucalyptol, o-cymene, octanal, dimethyl trisulfide, 1-nonanal, diallyl sulfide, decanal, linalool, β-caryophyllene, (E)-2-decenal, estragole, 1-dodecanal, anethole, geraniol, and eugenol greatly contribute (with OAV > 5) to the overall odor [39]. Only eucalyptol, linalool, and geraniol had OAVs >1 in each group, and the OAV values of these substances in groups B1 and B2 were low. The characteristic volatile substances of traditional beef bouillons were eucalyptol, 2-decanone, linalool, β-caryophyllene and geraniol. In particular, eucalyptol and linalool play the most important roles, and these substances mainly come from spices. Eucalyptol and linalool were also the key volatile substances in the two instant bouillons; however, the other three substances contributed less to flavor than they did in the traditional beef bouillons. From the instant bouillon data, the OAVs of the B2 group were close to those of the A1–A8 groups.

#### 3.1.3. Principal Component Analysis

**Figure 1:**
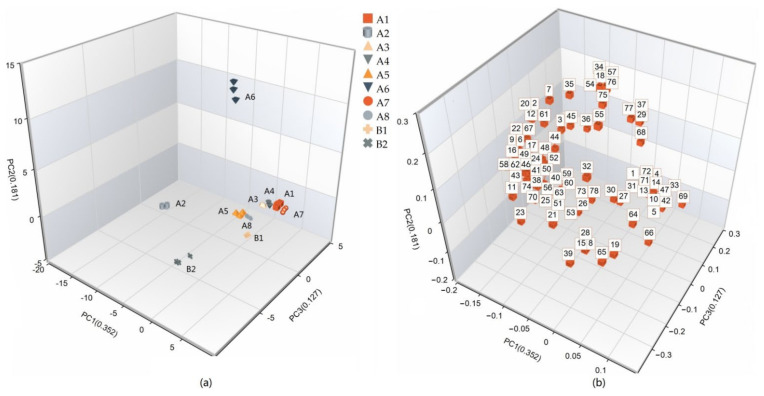
(**a**) Separation of the 10 groups in PCA space based on the OAV of volatile compounds; (**b**) Distribution of 78 volatile compounds in the 10 groups (numbers correspond to those in Table 3).

PCA is a multivariate statistical tool that can extract maximum information from the main influencing variables of the sample spatial distribution [9], and this method was used to explain the differences between samples. The OAVs of the 78 volatile compounds in Table 3 were analyzed by PCA, mainly because some substances with an OAV <1 still contributed to the flavor of the products, and the data were investigated by MSpectrum Pattern software after normalization. PC1 had an eigenvalue of 27.75, PC2 had an eigenvalue of 14.22, and PC3 had an eigenvalue of 10.03. These first three principal components explained 35.2%, 18.1% and 12.70% of the overall variance (Figure 1). The PCA plot includes these first three dimensions, which explain 66.00% of the total variation. As shown in the score plots (Figure 1a), the A2 and A6 groups were well differentiated from the other groups in the PCA plot. The B1 and B2 groups could not be well distinguished from the remaining groups in PC1 and PC2 but could be well differentiated in PC3. Since PC3 only explained 12.70% of the total variance, the difference between B2 and the other groups was not significant. The loading plot (Figure 1b) shows that the aroma components 1-methylnaphthalene (64) and o-anisaldehyde (66) could be the characteristic components of Group B1, and benzaldehyde (39), heptanal (15), 2-butylfuran (8), 2-pentylfuran (19), 2-nonanone (28) and 2-phenylethanol (65), which are common volatile substances of meat, could be the characteristic components of Group B2. The above substances may have caused the odor difference between the instant and traditional beef bouillons.

#### 3.1.4. Clustering Analysis

**Figure 2:**
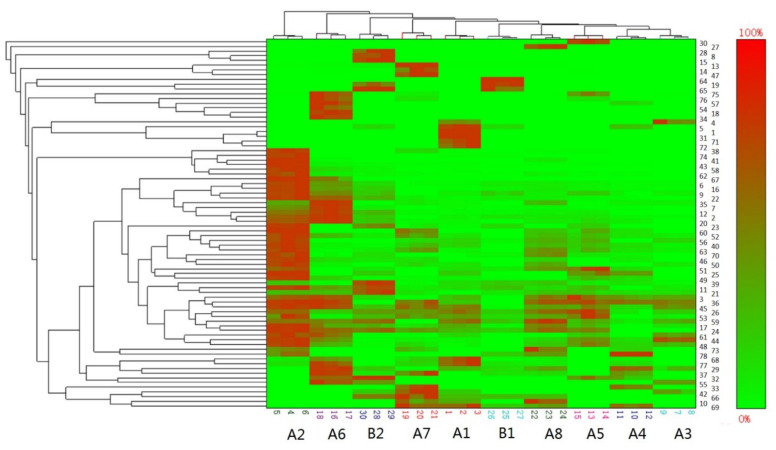
Cluster analysis heat map of volatile components (number on vertical axis correspond to those in Table 3).

Two-dimensional clustering analysis using the angle cosine method was used to analyze the differences among different groups and determine the degrees of homogeneity. The heat map (Figure 2) showed that according to the average distance between groups, B1 could be classified into one group with A3, A4, A5 and A8. When the average distance between groups increased, B2 could be clustered with other groups, except for A2 and A6. The results indicated that the difference between the instant and traditional beef bouillons was smaller than that among the traditional beef bouillons. In the heat map, the trend of data change can be seen intuitively through the color (green–red) scale. As can be seen from Figure 2, the composition of volatile substances of A2 and A6 and the other groups is more significantly different from that of the other groups. As can be seen from Figure 2, A2 and A6 have more obvious differences in volatile substance composition with other groups. Compared with other groups, the composition of volatile substances of B1 is mainly different from other groups in content rather than type. The difference between B2 and other groups may be due to the presence of 2-butylfuran (8), heptanal (15) and 2-nonanone (28), which cannot be found in other groups. 2-Butylfuran is the reaction product of 2, 4-decanodienal and cysteine. 2-Nonanone and heptanal are derived from the oxidation of saturated fatty acids. These three substances may be related to the use of concentrated beef bone broth in group B2 [31].

### 3.2. E-Nose Analysis

**Figure 3:**
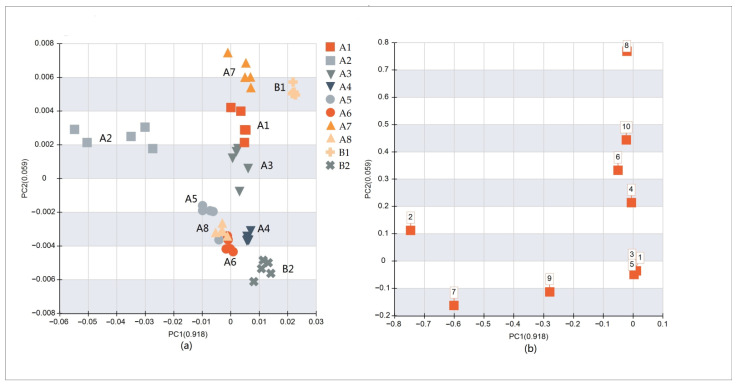
(**a**) Separation of the 10 groups in PCA space based on the data of E-nose; (**b**) distribution of 10 sensor responses in the 10 groups (numbers correspond to those in Table 1).

**Figure 4:**
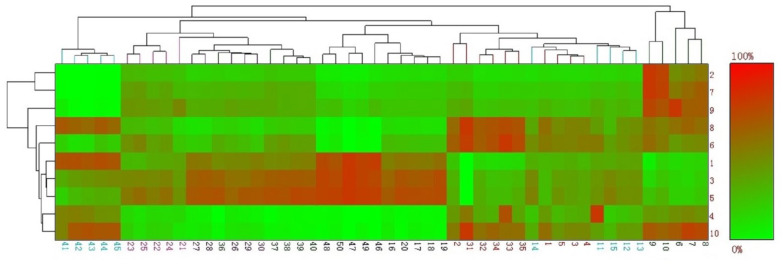
Cluster analysis heat map of E-nose (number on vertical axis correspond to those in Table 1, number on horizontal axis each five connected numbers correspond to a group, numbers 1–5 correspond to the five parallels of group A1, and so on).

The E-nose is an efficient tool to acquire comprehensive information associated with the volatile compounds in samples, and slight changes in volatile compounds can result in differences in sensor response. As shown in Table 5, the W5S and W1W sensors had strong responses to volatile compounds in the samples, and the response values of the two instant bouillons were lower than those of all traditional bouillons. Normally, W5S and W1W sensors are considered sensitive to nitrogen oxides and sulfides; however, the GC-MS results showed that the contents of these two substances were not high, which may be due to the nonspecific response of the sensors. However, according to the results from the 10 sensors, the response values of instant beef bouillons were lower than those of traditional beef bouillons. Except for the W5S, W1W, W2S and W2W sensors, there was little difference in response values among the sensors, which means that there was no obvious difference between the traditional and instant bouillons. As depicted in Figure 3, PC1 and PC2 explained 97.7% of the total variance, and PC1 explained 91.8%. From the score plot, B1 and B2 could not be distinguished from the other 7 groups, except for A2 on PC1. Group B1 was closer to groups A7 and A1 in the score plot space, while group B2 was closer to groups A4 and A6. B1, A1 and A7 were differentiated from the other groups by the W2S sensor and had positive score values on PC2. B2, A4 and A6 were differentiated from other groups by the W1C, W3C and W5C sensors and had negative score values on PC2. A2 was differentiated from the other groups by sensor W5S and was negatively correlated with PC1. Cluster analysis (Figure 4) showed that the flavor characteristics of B2 were close to those of most traditional beef bouillons, even though the difference between B1 and the other groups was smaller than that of A2.

### 3.3. E-Tongue Analysis

**Figure 5:**
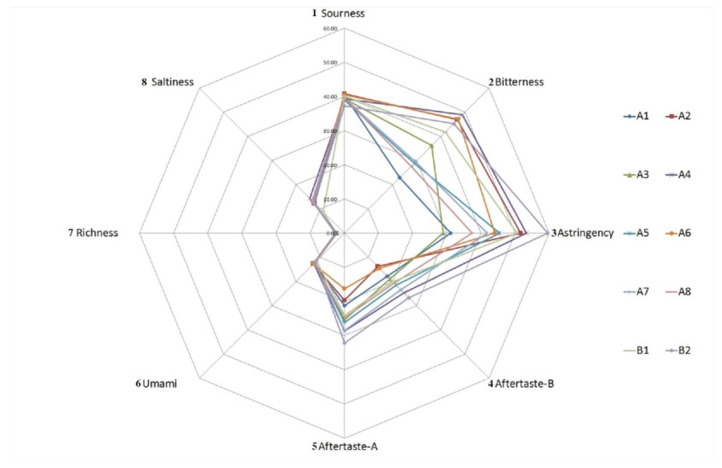
Radar chart of E-tongue data for beef bouillons.

**Figure 6:**
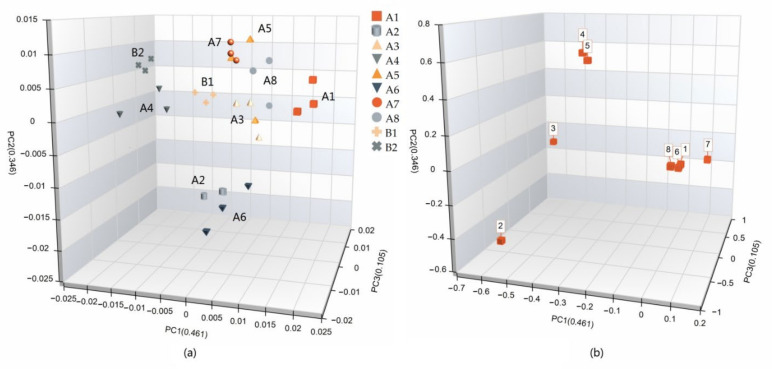
(**a**) Separation of the 10 groups in PCA space based on the data of E-tongue; (**b**) distribution of 8 sensor responses in the 10 groups (numbers correspond to those in Figure 5).

The E-tongue mimics human taste perception and can detect taste properties by electronic sensors. Figure 5 is the radar map of the beef bouillons determined by the E-tongue, and the results show that the richness (aftertaste–umami) of all beef bouillons was low (2.09–2.58), but the sourness, bitterness, astringency, aftertaste-B (aftertaste–bitterness) and aftertaste-A (aftertaste–astringency) were abnormally high. The umami and saltiness of group B1 and sourness of group B2 were the lowest among all groups. However, the aftertaste–astringency (aftertaste-A), aftertaste–bitterness (aftertaste-B) and astringency of group B2 were the highest; since this phenomenon did not appear in group B1, it may be due to the use of concentrated beef bone broth. In addition, the umami of both instant beef bouillons was lower than that of all traditional beef bouillons, which may be due to the destruction of peptides that produce umami taste during sterilization. According to the PCA, the three principal components explained 91.20% of the total variance. In the score plot (Figure 6a), B1 and B2 could be well differentiated from the other groups and were on the negative axis of PC1, the positive axis of PC2 (B1 was close to the coordinate axis), and the positive axis of PC3, with the traditional beef bouillons in other areas, suggesting that there were some differences in taste between the instant and traditional bouillons. As shown in Figure 6b (loading plot), PC1 was inversely related to bitterness, astringency, aftertaste-A and aftertaste-B, and it was positively related to umami (delicious taste). PC2 was positively related to aftertaste-A and aftertaste-B, and it was inversely related to bitterness and astringency. For PC3, there was a positive correlation with astringency and richness and a negative correlation with saltiness and bitterness.

### 3.4. Sensory Evaluation

The results of the sensory evaluation are shown in Table 6, and the results showed that the overall acceptability of instant bouillons (B1: 6.22, B2: 7.96) was in the range of acceptability of traditional bouillons (6.14–8.88). The overall acceptability of group B2 was only lower than that of groups A1 and A7, showing that B2 effectively recreates the flavor of traditional bouillons.

Three key aromas and five tastes of beef bouillons were evaluated by sensory evaluation. As shown in Table 5, the beef-like aroma in all bouillons was weak (from 2.60 to 4.70), consistent with the GC-MS results but inconsistent with the E-nose results. Groups B1 and B2 were fifth and ninth, respectively, among the 10 groups. The score of fat-like aroma in group A3 was the highest, with only 2.30, suggesting that the fat-like aroma in bouillons was less prominent than the beef-like aroma. There was no significant difference between most groups (*p* > 0.05). Different from the findings for the above two aromas, the bouillons had strong spice-like aromas (from 5.30 to 9.20), consistent with the GC-MS findings. Analysis of the results for the three kinds of aromas indicated that the scores for each index for the B2 group were in the middle to upper levels, which indicated that this treatment could recreate the flavor of traditional bouillons. The bouillons mainly reflected salty, umami and astringency tastes, while the scores of sour and bitter tastes were not high, inconsistent with the E-tongue results. This might be due to the sensors responding to other compounds in the bouillons that are not astringent, sour or bitter. In addition, the salty taste score of group B1 was significantly lower than those of the other groups (*p* < 0.05), and B2 had higher scores in both salty taste and umami taste.

## 4. Conclusions

In the present study, SPME-GC/MS combined with E-nose, E-tongue and sensory evaluation was used to investigate the volatile profiles and taste properties of traditional and instant Lanzhou beef bouillons. A total of 133 volatile compounds were identified; in terms of the total amount of volatile compounds, the instant beef bouillons ranked 10th and 8th (groups B1 and B2, respectively) among the 10 groups, and the volatile compound contents from spices were significantly lower than those from traditional sources. The characteristic volatile substances of traditional beef bouillons were eucalyptol, 2-decanone, linalool, β-caryophyllene and geraniol, especially eucalyptol and linalool. Eucalyptol and linalool were also the key volatile substances in the two instant bouillons, but their contents were lower, and there were fewer other key substances than in traditional bouillons. E-nose showed that the difference between instant and traditional beef bouillons was less than that between different traditional beef bouillons. The taste profiles of traditional and instant beef bouillons were significantly different according to E-tongue analysis. However, the sensory evaluation showed that spice-like aroma, salty taste and umami taste were the main flavor characteristics of beef bouillons, and B2 treatment recreated the flavor profiles of traditional bouillons. In general, the difference in aroma between traditional and instant beef bouillons may be due to the loss of volatile substances caused by the concentration process, but the reasons for taste differences are sterilization and the use of concentrated beef bone broth. Increasing the proportion of spices or using low temperature sterilization process may be useful to improve the quality of instant beef bouillons. In addition, the effect of concentrated beef bone broth on flavor, especially taste, is worth further exploration.

## Figures and Tables

**Table 1:** Information of 10 sensors for electronic nose.

No.	Sensor Name	Representative Material Species	Description(s)
1	W1C	Aromatic	Sensitive to aromatic constituents, benzene
2	W5S	Broad range	Sensitive to nitrogen oxides
3	W3C	Aromatic	Sensitive to aroma, ammonia
4	W6S	Hydrogen	Sensitive to hydrides
5	W5C	Arom-aliph	Sensitive to short-chain alkane aromatic component
6	W1S	Broad-methane	Sensitive to methyl
7	W1W	Sulphur-organic	Sensitive to sulfides
8	W2S	Broad-alcohol	Sensitive to alcohols, aldehydes and ketones
9	W2W	Sulf-chlor	Sensitive to organic sulfides
10	W3S	Methane-aliph	Sensitive to long-chain alkanes

**Table 2:** The corresponding references of odors and taste.

Attributes	Definition with Reference Material
Overall acceptability	-
Beef like	Cooked beef
Fat like	Warmed beef tallow
Spice like	0.3% Cinnamon oil solution in water
Salty taste	Sodium chloride 0.5% solution in water
Astringency taste	Aluminum sulfate 0.02‰ solution in water
Sour taste	Citric acid 0.3 g/L solution in water
Bitter taste	Quinine chloride 0.05% solution in water
Umami taste	Monosodium glutamate, 0.05% solution in water

**Table 3:** Identification and quantification of volatile compounds in each group (μg/kg).

^1^ Compound	Cas	^2^ A1	A2	A3	A4	A5	A6	A7	A8	B1	B2	RI	^3^ RI (Reference)
**Aldehyde**													
n-Pentanal	110-62-3	1.10 ± 0.10 ^a^	—	—	—	—	—	—	—	—	—	—	929
n-Hexanal	66-25-1	14.17 ± 0.48 ^a^	—	—	1.51 ± 0.05 ^b^	—	—	—	—	0.91 ± 0.04 ^c^	0.88 ± 0.07 ^c^	1079	1083
(E)-2-methyl-2-butenal	1115-11-3	3.03 ± 0.18 ^a^	—	—	—	—	—	—	—	—	—	1093	1095
Heptanal	111-71-7	—	—	—	—	—	—	—	—	—	0.51 ± 0.03 ^a^	1186	1188
Octanal	124-13-0	3.54 ± 0.18 ^e^	3.39 ± 0.05 ^a^	—	—	2.05 ± 0.12 ^c^	2.59 ± 0.07 ^b^	1.66 ± 0.15 ^d^	2.43 ± 0.23 ^b^	0.86 ± 0.06 ^f^	2.44 ± 0.20 ^b^	1288	1287
1-Nonanal	124-19-6	11.40 ± 0.75 ^c,d^	—	3.12 ± 1.18 ^d,e^	8.07 ± 0.33 ^b^	—	9.96 ± 0.21 ^a^	—	4.60 ± 0.57 ^c^	1.79 ± 0.15 ^f^	2.73 ± 0.20 ^e^	1393	1390
(E)-2-octenal	2548-87-0	—	—	—	—	0.88 ± 0.05 ^a^	—	—	—	—	—	1430	1434
Decanal	112-31-2	—	—	—	2.96 ± 0.48 ^c^	—	6.34 ± 0.18 ^a^	4.93 ± 1.35 ^b^	1.08 ± 0.12 ^d^	0.92 ± 0.11 ^d^	0.79 ± 0.07 ^d^	1498	1498
Benzaldehyde	100-52-7	72.27 ± 1.93 ^c^	42.08 ± 0.73 ^b^	5.58 ± 0.30 ^e^	18.72 ± 0.63 ^c,d^	12.65 ± 1.86 ^d^	1.33 ± 0.15 ^e^	3.89 ± 0.81 ^e^	40.75 ± 2.79 ^b^	36.54 ± 1.30 ^b^	140.38 ± 8.61 ^a^	1526	1527
(E)-2-decenal	3913-81-3	—	9.42 ± 0.38 ^a^	—	0.97 ± 0.01 ^e^	—	1.68 ± 0.15 ^c^	1.46 ± 0.18 ^c,d^	2.01 ± 0.08 ^b^	—	1.26 ± 0.08 ^d,e^	1644	1642
(Z)-citral	106-26-3	7.59 ± 0.66 ^c^	19.33 ± 0.85 ^a^	2.42 ± 0.18 ^c^	—	—	1.28 ± 0.14 ^d^	3.31 ± 0.88 ^c^	8.28 ± 0.64 ^b^	—	—	1682	1688
1-Dodecanal	112-54-9	—	—	—	—	—	3.06 ± 0.52 ^a^	—	—	—	—	1711	1709
(E)-citral	141-27-5	17.77 ± 1.70 ^c^	27.88 ± 1.61 ^a^	6.10 ± 0.31 ^c^	—	10.44 ± 2.25 ^b^	—	4.78 ± 0.61 ^c^	11.63 ± 0.63 ^b^	1.32 ± 0.07 ^e^	—	1734	1733
4-Isopropylbenzaldehyde	122-03-2	16.58 ± 1.43 ^b^	5.43 ± 0.18 ^b^	3.11 ± 0.24 ^d^	0.87 ± 0.07 ^f^	3.19 ± 0.51 ^d^	4.38 ± 0.28 ^c^	—	7.32 ± 0.50 ^a^	2.24 ± 0.17 ^e^	5.61 ± 0.41 ^b^	1782	1785
Trans-2-dodecenal	4826-62-4	—	—	—	3.48 ± 0.28 ^a^	—	1.96 ± 0.25 ^b^	—	1.26 ± 0.14 ^c^	—	—	1854	1849
4-Propylbenzaldehyde	28785-06-0	14.25 ± 0.95 ^d,e^	21.32 ± 0.87 ^b^	3.86 ± 0.26 ^e^	0.98 ± 0.06 ^f^	—		6.73 ± 2.00 ^d^	19.06 ± 1.30 ^c^	1.20 ± 0.11 ^f^	31.53 ± 1.94 ^a^	1871	—
o-Anisaldehyde	135-02-4	4.02 ± 0.95 ^c^	—	—	—	—	—	4.60 ± 0.96 ^a^	1.16 ± 0.09 ^c^	1.06 ± 0.08 ^c^	3.12 ± 0.13 ^b^	1966	1941
2-Propenal	101-39-3	6.87 ± 0.71 ^e^	16.32 ± 0.17 ^a^	2.72 ± 0.20 ^e^	0.94 ± 0.06 ^f^	3.58 ± 0.59 ^e^	5.92 ± 0.72 ^d^	2.40 ± 0.27 ^e^	11.40 ± 1.04 ^c^	3.39 ± 0.42 ^e^	12.81 ± 1.29 ^b^	1984	1992
4-Methoxybenzaldehyde	123-11-5	5.35 ± 0.53 ^a^	—	—	1.49 ± 0.14 ^b^	—	—	1.81 ± 0.31 ^a^	0.97 ± 0.14 ^c^	0.55 ± 0.06 ^e^	—	2029	2027
Cinnamaldehyde	14371-10-9	86.22 ± 10.13 ^e,f^	1010.89 ± 78.50 ^a^	21.44 ± 2.00 ^e,f^	70.35 ± 7.13 ^d,e^	108.51 ± 10.94 ^d^	26.05 ± 3.43 ^e,f^	5.04 ± 1.08 ^f^	610.60 ± 30.81 ^b^	42.80 ± 7.12 ^e,f^	183.05 ± 17.62 ^c^	2044	2040
**Alkane**													
n-Decane	124-18-5	—	—	—	0.97 ± 0.14 ^c^	—	2.08 ± 0.06 ^a^	—	1.41 ± 0.14 ^b^	1.44 ± 0.05 ^b^	—	—	980
**Alkenes**													
2-Pinene	80-56-8	5.55 ± 1.12 ^e^	22.94 ± 0.92 ^b^	0.81 ± 0.04 ^f^	1.44 ± 0.13 ^e,f^	3.70 ± 0.17 ^d^	27.68 ± 0.48 ^a^	—	3.28 ± 0.32 ^d^	3.76 ± 0.21 ^d^	7.02 ± 0.37 ^c^	1014	1015
3-Thujene	2867-05-2	2.61 ± 0.20 ^d^	4.13 ± 0.06 ^b^	—	—	—	5.02 ± 0.02 ^a^	—	—	—	1.08 ± 0.10 ^c^	1021	1022
Comphene	79-92-5	4.46 ± 1.05 ^d^	12.42 ± 0.61 ^a^	—	—	1.79 ± 0.20 ^d^	4.39 ± 0.46 ^b^	—	1.63 ± 0.12 ^d^	3.37 ± 0.25 ^c^	4.80 ± 0.38 ^b^	1055	1060
β-Pinene	127-91-3	5.12 ± 0.82 ^e,f^	38.29 ± 1.79 ^a^	1.23 ± 0.04 ^f^	0.96 ± 0.08 ^f^	2.96 ± 0.40 ^e^	20.47 ± 0.38 ^b^	0.74 ± 0.27 ^f^	4.52 ± 0.21 ^d^	4.38 ± 0.23 ^d^	7.26 ± 0.39 ^c^	1090	1094
Sabinen	3387-41-5	5.67 ± 0.80 ^d^	25.76 ± 1.04 ^b^	—	0.80 ± 0.05 ^d^	0.91 ± 0.13 ^d^	45.11 ± 1.33 ^a^	—	1.85 ± 0.24 ^d^	0.81 ± 0.04 ^d^	3.45 ± 0.21 ^c^	1107	1109
3-Carene	13466-78-9	23.96 ± 3.39 ^e^	86.19 ± 3.86 ^a^	6.55 ± 0.57 ^e,f^	3.80 ± 0.44 ^f,g^	24.61 ± 1.80 ^c^	54.89 ± 1.23 ^b^	1.04 ± 0.21 ^g^	10.03 ± 0.77 ^e^	19.44 ± 0.73 ^d^	26.15 ± 1.68 ^c^	1132	1135
α-Phellandrene	99-83-2	8.72 ± 0.45 ^e,f^	13.27 ± 0.49 ^a^	2.13 ± 0.25 ^e,f^	0.72 ± 0.10 ^g^	4.90 ± 0.49 ^d^	7.11 ± 0.59 ^c^	2.06 ± 0.27 ^e,f^	2.97 ± 0.19 ^e^	4.66 ± 0.07 ^d^	11.91 ± 0.76 ^b^	1152	1155
β-Myrcene	123-35-3	9.15 ± 0.54 ^e^	69.03 ± 2.00 ^b^	—	—	8.88 ± 0.57 ^d^	94.90 ± 4.07 ^a^	3.91 ± 0.23 ^e^	7.92 ± 0.46 ^d^	3.61 ± 0.22 ^e^	34.99 ± 2.60 ^c^	1156	1160
α-Terpilene	99-86-5	5.89 ± 0.76 ^e^	3.91 ± 0.17 ^d^	1.05 ± 0.14 ^f^	—	—	6.11 ± 0.21 ^c^	241.31 ± 10.83 ^a^	1.35 ± 0.02 ^e,f^	3.31 ± 0.13 ^d^	11.48 ± 0.80 ^b^	1168	1172
Limonene	138-86-3	23.48 ± 1.83 ^g,h^	314.22 ± 10.35 ^a^	8.32 ± 0.69 ^g,h^	12.36 ± 0.76 ^f,g^	23.08 ± 1.52 ^e^	156.00 ± 5.89 ^b^	2.45 ± 0.17 ^h^	36.76 ± 3.23 ^d^	19.91 ± 0.38 ^e,f^	78.46 ± 5.09 ^c^	1188	1186
β-Phellandrene	555-10-2	—	—	—	—	—	63.26 ± 2.72 ^a^	—	—	—	—	1207	1208
(Z)-β-ocimene	3338-55-4	—	23.83 ± 0.79 ^b^	0.82 ± 0.07 ^d^	2.18 ± 0.18 ^c,d^	3.50 ± 0.15 ^c^	44.95 ± 1.29 ^a^	1.35 ± 0.25 ^d^	3.77 ± 0.45 ^c^	0.51 ± 0.01 ^d^	24.98 ± 2.01 ^b^	1234	1234
γ-Terpinene	99-85-4	10.32 ± 0.36 ^f,g^	59.72 ± 1.47 ^b^	—	5.86 ± 0.50 ^e,f^	9.82 ± 3.25 ^d^	69.57 ± 1.47 ^a^	2.51 ± 0.38 ^g^	7.57 ± 0.51 ^d,e^	—	19.50 ± 1.33 ^c^	1239	1238
β-Ocimene	13877-91-3	2.00 ± 0.10 ^f^	39.06 ± 1.51 ^a^	—	2.50 ± 0.21 ^e^	4.98 ± 0.85 ^d^	24.38 ± 0.88 ^b^	2.34 ± 0.45 ^e^	4.75 ± 0.41 ^d^	—	13.18 ± 1.04 ^c^	1249	1251
Styrene	100-42-5	2.39 ± 0.13 ^e^	5.23 ± 0.28 ^b^	2.87 ± 0.22 ^d^	2.82 ± 0.17 ^d^	4.06 ± 0.83 ^c^	4.85 ± 0.11 ^b,c^	2.39 ± 0.42 ^d^	4.34 ± 0.36 ^b,c^	2.21 ± 0.11 ^d^	12.99 ± 1.18 ^a^	1257	1254
Terpinolene	586-62-9	1.82 ± 0.02 ^d^	15.05 ± 0.37 ^a^	—	—	3.45 ± 0.22 ^c^	—	1.05 ± 0.87 ^d^	—	2.78 ± 0.06 ^c^	9.30 ± 0.72 ^b^	1277	1279
Perillene	539-52-6	—	—	—	—	—	—	—	—	1.49 ± 0.15 ^a^	1.59 ± 0.14 ^a^	1422	1431
Dehydro-p-cymene	1195-32-0	—	—	—	1.21 ± 0.14 ^c^	2.22 ± 0.29 ^b^	2.24 ± 0.10 ^b^	—	—	—	3.45 ± 0.18 ^a^	1443	1438
α-Cubebene	17699-14-8	3.36 ± 0.13 ^e^	8.15 ± 0.48 ^a^	4.18 ± 0.14 ^c^	2.73 ± 0.23 ^d^	2.31 ± 0.18 ^d^	6.85 ± 0.42 ^b^	—	2.68 ± 0.21 ^d^	—	0.62 ± 0.05 ^f^	1452	1453
p-Menth-3-ene	20307-84-0	13.88 ± 0.65 ^e^	41.92 ± 1.86 ^a^	36.45 ± 2.61 ^b^	12.02 ± 1.04 ^c^	9.62 ± 0.84 ^c,d^	7.74 ± 0.78 ^d^	2.32 ± 0.29 ^e^	10.73 ± 1.20 ^c^	3.54 ± 0.10 ^e^	3.81 ± 0.28 ^e^	1466	1467
(+)-Cyclosativene	22469-52-9	—	8.00 ± 0.40 ^a^	1.09 ± 0.09 ^e^	5.29 ± 0.44 ^c^	6.11 ± 0.32 ^b^	—	—	3.74 ± 0.21 ^d^	—	—	1475	1477
Ylangene	14912-44-8	—	3.36 ± 0.15 ^a^	—	1.15 ± 0.07 ^c^	0.88 ± 0.05 ^d^	—	—	1.43 ± 0.04 ^b^	—	—	1478	1481
Copaene	3856-25-5	18.06 ± 0.13 ^f^	96.03 ± 4.62 ^b^	57.77 ± 3.14 ^d^	125.69 ± 10.07 ^a^	19.42 ± 1.01 ^e^	25.28 ± 2.25 ^e^	2.91 ± 0.16 ^f^	75.66 ± 5.70 ^c^	3.79 ± 0.19 ^f^	3.94 ± 0.36 ^f^	1485	1488
β-Cubebene	13744-15-5	—	6.07 ± 0.21 ^a^	2.22 ± 0.12 ^b^	—	—	—	—	—	—	—	1535	1537
Trans-α-bergamotene	13474-59-4	—	—	—	1.30 ± 0.13 ^b^	2.41 ± 0.32 ^a^	—	—	—	—	—	1556	1561
Isocaryophyllene	118-65-0	—	—	13.14 ± 0.81 ^a^	1.98 ± 0.05 ^c,d^	2.05 ± 0.34 ^c,d^	1.52 ± 0.11 ^d,e^	3.36 ± 0.28 ^b^	2.64 ± 0.21 ^c^	—	1.30 ± 0.10 ^e^	1572	1572
β-Elemene	515-13-9	5.42 ± 0.89 ^f^	17.25 ± 0.53 ^a^	9.67 ± 0.53 ^c^	7.58 ± 0.55 ^d^	17.19 ± 1.51 ^a^	11.98 ± 0.97 ^b^	1.63 ± 0.44 ^f^	4.46 ± 0.38 ^e^	1.08 ± 0.02 ^f^	3.30 ± 0.25 ^e^	1587	1586
β-Caryophyllene	87-44-5	192.43 ± 20.52 ^e,f^	802.49 ± 18.77 ^a^	695.22 ± 37.07 ^b^	151.39 ± 10.43 ^d^	458.50 ± 17.35 ^c^	463.56 ± 46.55 ^c^	76.95 ± 9.20 ^e^	173.35 ± 12.14 ^d^	21.21 ± 0.48 ^f^	35.84 ± 1.97 ^e,f^	1592	1588
(−)-Alloaromadendrene	25246-27-9	3.14 ± 0.54 ^d^	2.20 ± 0.05 ^c^	—	2.81 ± 0.18 ^b^	5.22 ± 0.79 ^a^	1.97 ± 0.24 ^c^	1.23 ± 0.21 ^d^	—	—	—	1640	1642
(+)-Aromadendrene	489-39-4	—	16.68 ± 0.81 ^a^	—	—	—	—	2.01 ± 0.41 ^c^	4.45 ± 0.30 ^b^	—	1.20 ± 0.06 ^d^	1641	1637
Humulene	6753-98-6	9.63 ± 2.65 ^f^	53.46 ± 2.26 ^a^	20.12 ± 1.09 ^c^	12.71 ± 0.65 ^d,e^	33.77 ± 4.72 ^b^	21.67 ± 2.43 ^c^	10.06 ± 2.69 ^e^	14.78 ± 1.32 ^d^	1.63 ± 0.02 ^f^	2.31 ± 0.15 ^f^	1665	1673
γ-Muurolene	30021-74-0	8.88 ± 2.38 ^e^	44.92 ± 1.49 ^a^	—	10.09 ± 0.53 ^c^	10.20 ± 3.18 ^c^	6.36 ± 0.34 ^d^	—	16.93 ± 1.31 ^b^	2.24 ± 0.08 ^e^	2.97 ± 0.16 ^e^	1685	1681
Guaiene	88-84-6	7.75 ± 0.71 ^c^	14.36 ± 0.99 ^a^	—	3.02 ± 0.31 ^c^	15.18 ± 2.90 ^a^	—	—	9.01 ± 0.63 ^b^	1.94 ± 0.06 ^c^	2.06 ± 0.09 ^c^	1687	1671
Germacrene D	23986-74-5	—	12.26 ± 0.48 ^a^	—	3.89 ± 0.29 ^c^	4.21 ± 0.14 ^c^	6.22 ± 0.66 ^b^	—	2.48 ± 0.22 ^d^	—	1.36 ± 0.05 ^e^	1705	1710
β-Cadinene	523-47-7	—	—	—	—	4.24 ± 0.44 ^a^	—	—	—	—	—	1709	—
Borneol	507-70-0	37.20 ± 3.11 ^b,c^	20.24 ± 5.57 ^a^	2.17 ± 0.16 ^e^	—	20.60 ± 3.80 ^a^	0.83 ± 0.01 ^e^	13.59 ± 1.75 ^b^	11.08 ± 0.72 ^b,c^	4.37 ± 0.17 ^d,e^	8.24 ± 0.17 ^c,d^	1700	1700
Longifolene-(v4)	61262-67-7	18.77 ± 3.16 ^e^	49.54 ± 2.21 ^a^	10.20 ± 0.59 ^d^	16.82 ± 1.32 ^c^	40.86 ± 4.86 ^b^	—	4.16 ± 0.88 ^e^	16.90 ± 1.15 ^c^	3.12 ± 0.15 ^e^	3.56 ± 0.34 ^e^	1713	—
Eremophilene	10219-75-7	—	—	—	—	—	8.83 ± 1.07 ^a^	—	—	—	—	1715	1710
Zingiberene	495-60-3	82.36 ± 16.43 ^c,d^	87.02 ± 3.53 ^b^	17.71 ± 1.28 ^c,d^	34.69 ± 2.58 ^c,d^	286.21 ± 38.44 ^a^	10.00 ± 1.06 ^d^	22.27 ± 3.95 ^c,d^	41.26 ± 2.07 ^c^	18.63 ± 0.42 ^c,d^	28.79 ± 2.28 ^c,d^	1719	1713
α-Muurolene	10208-80-7	—	27.00 ± 1.27 ^a^	—	—	—	—	—	—	—	—	1722	1720
β-Bisabolene	495-61-4	84.82 ± 11.50 ^c^	125.04 ± 4.82 ^a^	—	44.65 ± 2.76 ^c^	104.15 ± 10.82 ^b^	—	25.04 ± 2.34 ^c^	23.21 ± 1.58 ^c^	12.38 ± 0.09 ^d^	—	1725	1722
Azulene	275-51-4	3.99 ± 0.51 ^b^	—	—	—	—	—	1.95 ± 0.25 ^a^	—	1.24 ± 0.14 ^b^	—	1743	1746
(Z,E)-α-farnesene	26560-14-5	15.59 ± 3.50 ^c^	28.35 ± 1.04 ^b^	2.06 ± 0.25 ^d,e^	11.15 ± 0.52 ^c^	36.98 ± 7.72 ^a^	3.75 ± 0.47 ^d,e^	3.37 ± 1.08 ^d,e^	7.55 ± 0.45 ^c,d^	1.32 ± 0.03 ^e^	2.08 ± 0.11 ^d,e^	1749	1721
(+)-δ-Cadinene	483-76-1	19.14 ± 8.04 ^d^		11.15 ± 0.51 ^c,d^	23.21 ± 1.08 ^c^	34.54 ± 7.62 ^b^	—	13.44 ± 3.00 ^c^	43.03 ± 3.25 ^a^	5.61 ± 0.11 ^d^	—	1755	1753
Sesquiphellandrene	20307-83-9	69.90 ± 14.66 ^c,d e^	104.50 ± 4.68 ^b^	5.73 ± 0.38 ^e^	37.61 ± 2.51 ^c^	129.88 ± 22.84 ^a^	10.19 ± 1.18 ^d,e^	24.47 ± 2.72 ^c,d^	30.84 ± 2.12 ^c^	7.30 ± 0.20 ^d,e^	9.85 ± 0.50 ^d,e^	1768	1764
α-Curcumene	644-30-4	97.18 ± 16.75 ^d,e^	193.35 ± 7.60 ^a^	9.86 ± 0.67 ^e^	69.85 ± 4.65 ^b^	194.58 ± 29.70 ^a^	25.71 ± 2.72 ^d,e^	41.94 ± 1.63 ^c,d^	62.07 ± 3.90 ^b,c^	17.30 ± 0.13 ^e^	19.06 ± 1.62 ^e^	1773	1770
Germacrene b	15423-57-1	—	—	—	—	2.61 ± 0.40 ^a^	—	—	1.02 ± 0.11 ^b^	—	—	1825	1823
α-Calacorene	21391-99-1	—	3.70 ± 0.08 ^a^	—	—	—	—	—	—	—	—	1915	1916
Caryophyllene oxide	1139-30-6	2.33 ± 0.34 ^c^	6.32 ± 0.28 ^a^	0.87 ± 0.06 ^c^	—	—	3.16 ± 0.38 ^b^	—	—	0.61 ± 0.07 ^c^	0.73 ± 0.07 ^c^	1979	1976
1-Methylnaphthalene	90-12-0	—	—	—	—	—	—	—	—	0.65 ± 0.09 ^a^	—	1888	1891
**Aromatic**													
Toluene	108-88-3	2.47 ± 0.09 ^c,d^	2.09 ± 0.07 ^a,b^	1.20 ± 0.05 ^c^	1.18 ± 0.15 ^c^	2.00 ± 0.52 ^a,b^	2.27 ± 0.13 ^a^	—	1.75 ± 0.24 ^b^	—	0.54 ± 0.03 ^d^	1037	1043
Ethylbenzene	100-41-4	—	2.61 ± 0.09 ^b^	1.84 ± 0.12 ^b^	2.40 ± 0.03 ^b^	2.94 ± 0.37 ^b^	3.80 ± 0.30 ^b^	390.06 ± 51.23 ^a^	2.32 ± 0.14 ^b^	—	—	1181	1175
o-Cymene	527-84-4	7.85 ± 0.50 ^f,g^	31.95 ± 1.02 ^a^	1.53 ± 0.09 ^g^	2.03 ± 0.15 ^g^	2.69 ± 0.19 ^f,g^	26.28 ± 0.35 ^b^	3.54 ± 0.83 ^e,f^	3.99 ± 0.27 ^d,e^	4.78 ± 0.21 ^d^	13.20 ± 0.90 ^c^	1266	1274
Elemicin	487-11-6	16.04 ± 2.44 ^a^	3.93 ± 0.10 ^b^	—	1.10 ± 0.10 ^d^	—	3.41 ± 0.56 ^b,c^	3.19 ± 0.33 ^c^	—	—	0.82 ± 0.06 ^d^	2230	2232
**Sulfide**													
Dimethyl disulfide	624-92-0	5.91 ± 0.34 ^b^	—	2.63 ± 0.75 ^a^	—	—	—	—	—	—	—	1070	1071
**Heterocyclic**													
2-Butylfuran	4466-24-4	—	—	—	—	—	—	—	—	—	1.05 ± 0.09 ^a^	1132	1130
2-Pentylfuran	3777-69-3	—	—	—	—	—	—	—	—	0.81 ± 0.04 ^a^	0.55 ± 0.08 ^b^	1233	1229
1,3-Dithiane	505-23-7	21.09 ± 0.29 ^c^	—	—	—	—	15.54 ± 0.19 ^a^	5.60 ± 0.65 ^d^	11.92 ± 0.72 ^b^	—	—	1280	1296
2-Hexylfuran	3777-70-6	—	—	—	—	—	—	—	—	—	2.19 ± 0.25 ^a^	1332	1329
Linalyl oxide	5989-33-3	2.30 ± 0.32 ^a^	—	—	—	—	—	—	—	—	—	1441	1443
Cosmene	460-01-5	—	—	—	—	—	1.89 ± 0.14 ^a^	—	—	—	—	1452	1460
**Ethers**													
Diallyl sulfide	592-88-1	1.97 ± 0.08 ^c^	—	—	—	—	—	1.00 ± 0.17 ^b^	1.44 ± 0.03 ^a^	—	—	1141	1148
Dimethyl trisulfide	3658-80-8	—	—	—	—	—	—	—	0.96 ± 0.09 ^a^	—	—	1380	1376
Diallyl disulfide	2179-57-9	37.10 ± 2.19 ^e^	110.96 ± 0.85 ^b^	21.50 ± 0.58 ^d^	19.58 ± 1.05 ^d^	2.80 ± 0.26 ^f^	252.00 ± 5.33 ^a^	25.05 ± 5.56 ^d^	88.14 ± 5.69 ^c^	—	—	1481	1480
Estragole	140-67-0	33.68 ± 1.48 ^e^	166.66 ± 4.52 ^a^		29.13 ± 1.96 ^d^	53.89 ± 9.02 ^b^	41.00 ± 2.96 ^c^	6.41 ± 0.77 ^e,f^	—	2.58 ± 0.31 ^f^	2.22 ± 0.04 ^f^	1672	1671
Diallyl trisulfide	2050-87-5	38.16 ± 2.36 ^c^	60.73 ± 1.76 ^a^	6.02 ± 0.47 ^d^	4.69 ± 0.29 ^d,e^	1.60 ± 0.35 ^f^	—	2.26 ± 0.27 ^e,f^	37.46 ± 3.78 ^b^	—	—	1790	1789
Anethole	104-46-1	26.19 ± 1.25 ^c,d^	364.58 ± 26.66 ^a^	4.99 ± 0.86 ^d^	12.23 ± 1.19 ^c,d^	11.37 ± 1.04 ^c,d^	40.32 ± 5.67 ^b^	12.80 ± 1.43 ^c,d^	27.56 ± 5.97 ^b,c^	9.71 ± 1.55 ^c,d^	23.61 ± 2.98 ^b,c d^	1830	1834
Methylisoeugenol	93-16-3	8.39 ± 1.35 ^a^	—	—	—	—	2.71 ± 0.50 ^a^	0.78 ± 0.07 ^b^	0.84 ± 0.05 ^b^	—	—	2186	2185
Myristicin	607-91-0	31.11 ± 4.14 ^a^	1.89 ± 0.01 ^b,c^	—	1.61 ± 0.10 ^b,c^	—	10.11 ± 1.13 ^a^	2.51 ± 0.13 ^b^	0.95 ± 0.16 ^c^	1.34 ± 0.04 ^b,c^	—	2267	2257
**Alcohols**													
Eucalyptol	470-82-6	342.20 ± 21.98 ^c,d^	249.86 ± 2.90 ^a^	52.20 ± 1.83 ^e,f^	22.93 ± 1.18 ^g^	96.20 ± 12.46 ^d^	135.62 ± 30.74 ^c^	58.34 ± 10.23 ^e^	165.33 ± 4.28 ^b^	28.78 ± 0.69 ^f,g^	103.08 ± 13.46 ^d^	1199	1199
2-Heptanol	543-49-7	2.04 ± 0.16 ^c^	2.78 ± 0.10 ^a^	—	1.11 ± 0.07 ^b^	1.29 ± 0.29 ^b^	—	—	—	—	0.49 ± 0.01 ^c^	1320	1319
1-Octen-3-ol	3391-86-4	—	—	—	0.87 ± 0.08 ^b^	—	—	1.10 ± 0.19 ^a^	—	—	—	1453	1456
Cis-(+/-)-4-Thujanol	15537-55-0	13.03 ± 1.40 ^d^	14.29 ± 0.35 ^b^	1.35 ± 0.09 ^e^	—	—	19.86 ± 0.49 ^a^	—	6.47 ± 0.30 ^c^	—	—	1464	1469
2-Ethylhexan-1-ol	104-76-7	3.17 ± 0.36 ^a^	—	—	—	—	—	—	—	—	—	1490	1491
Linalool	78-70-6	606.79 ± 38.51 ^c^	886.80 ± 26.90 ^a^	51.11 ± 2.08 ^d,e^	163.34 ± 3.52 ^c^	267.01 ± 29.23 ^b^	66.75 ± 3.54 ^d^	270.53 ± 61.92 ^b^	306.29 ± 13.14 ^b^	7.43 ± 0.15 ^e^	32.81 ± 1.44 ^d,e^	1549	1552
1-Octanol	111-87-5	2.13 ± 0.20 ^d^	—	1.60 ± 0.15 ^b^	—	—	—	3.67 ± 0.41 ^a^	—	—	1.30 ± 0.08 ^c^	1558	1555
2-Cyclohexen-1-ol	29803-81-4	24.44 ± 2.05 ^b^	6.60 ± 0.25 ^c^	6.54 ± 0.39 ^c^	2.31 ± 0.11 ^f^	5.04 ± 0.56 ^d^	11.75 ± 0.54 ^a^	2.65 ± 0.31 ^f^	4.16 ± 0.45 ^e^	—	3.82 ± 0.19 ^e^	1562	1557
Terpinen-4-ol	562-74-3	397.81 ± 31.89 ^b,c^	198.66 ± 7.06 ^a^	120.39 ± 6.49 ^c,d^	48.95 ± 1.14 ^f^	116.44 ± 19.79 ^c,d^	189.98 ± 11.42 ^a^	150.89 ± 15.92 ^b^	101.39 ± 5.30 ^d^	15.70 ± 0.43 ^g^	71.11 ± 2.95 ^e^	1601	1606
1-Nonanol	143-08-8	—	—	—	—	—	—	2.04 ± 0.29 ^a^	—	—	—	1663	1666
α-Terpineol	98-55-5	138.41 ± 13.74 ^d^	253.68 ± 15.58 ^a^	36.22 ± 2.17 ^d^	50.03 ± 1.30 ^d^	84.38 ± 15.37 ^c^	100.72 ± 8.06 ^c^	138.18 ± 19.49 ^b^	96.46 ± 6.05 ^c^	14.75 ± 0.35 ^e^	40.89 ± 1.98 ^d^	1697	1697
(+)-Trans-piperitenol	16721-39-4	4.47 ± 0.75 ^c^	2.04 ± 0.17 ^b^	1.91 ± 0.12 ^b^	—	—	2.47 ± 0.17 ^a^	—	—	—	1.06 ± 0.12 ^d^	1745	1742
Nerol	106-25-2	—	19.33 ± 0.47 ^a^	1.05 ± 0.17 ^e^	4.18 ± 0.18 ^d^	6.73 ± 1.91 ^c^	—	13.24 ± 0.88 ^b^	6.25 ± 0.51 ^c^	—	0.70 ± 0.06 ^e^	1797	1794
(−)-Trans-carveol	1197-07-5	—	—	0.61 ± 0.06 ^a^	—	—	—	—	—	—	—	1836	1836
Geraniol	106-24-1	15.94 ± 2.49 ^d,e^	65.49 ± 3.29 ^a^	6.75 ± 0.57 ^d,e^	10.33 ± 0.37 ^d^	23.52 ± 6.23 ^c^	2.86 ± 0.34 ^e^	33.05 ± 5.48 ^b^	37.12 ± 2.39 ^b^	1.26 ± 0.03 ^e^	7.53 ± 0.53 ^d,e^	1848	1853
2-(4-Methylphenyl)propan-2-ol	1197-01-9	5.16 ± 0.64 ^a^	—	1.22 ± 0.15 ^b^	—	—	1.58 ± 0.05 ^a^	—	—	—	—	1852	1844
2-Phenylethanol	60-12-8	—	—	—	—	—	—	—	—	1.42 ± 0.07 ^b^	1.81 ± 0.14 ^a^	1913	1912
Cinnamyl alcohol	104-54-1	—	1.97 ± 0.04 ^b^	—	3.22 ± 0.19 ^a^	—	—	—	1.42 ± 0.05 ^c^	0.67 ± 0.05 ^d^	—	2286	2286
**Ketone**													
6-Methyl-5-hepten-2-one	110-93-0	12.68 ± 0.49 ^b^	4.99 ± 0.06 ^a^	2.23 ± 0.06 ^d^	1.06 ± 0.06 ^e^	5.33 ± 0.45 ^a^	2.22 ± 0.04 ^d^	3.36 ± 0.24 ^c^	4.52 ± 0.27 ^b^	2.27 ± 0.03 ^d^	3.26 ± 0.10 ^c^	1338	1341
2-Nonanone	821-55-6	—	—	—	—	—	—	—	—	—	1.12 ± 0.11 ^a^	1389	1386
2-Decanone	693-54-9	9.15 ± 0.28 ^d^	11.04 ± 0.17 ^a^	7.21 ± 0.37 ^c^	7.94 ± 0.41 ^b,c^	9.18 ± 0.97 ^b^	10.60 ± 0.57 ^a^	7.88 ± 1.42 ^b,c^	8.99 ± 0.73 ^b^	—	—	1493	1493
Camphor	76-22-2	5.47 ± 0.18 ^c^	107.67 ± 3.25 ^a^	—	—	1.85 ± 0.19 ^c^	—	20.66 ± 1.19 ^b^	1.90 ± 0.05 ^c^	—	1.03 ± 0.03 ^c^	1499	1499
4-Isopropyl-2-cyclohexenone	500-02-7	14.27 ± 2.39 ^b^	—	7.30 ± 0.75 ^a^	—	—	5.20 ± 0.43 ^b^	1.84 ± 0.40 ^d^	2.76 ± 0.21 ^c^	—	2.27 ± 0.17 ^c,d^	1669	1669
Piperitone	89-81-6	—	—	21.85 ± 1.37 ^b^	—	—	35.70 ± 3.85 ^a^	—	—	—	19.89 ± 1.90 ^b^	1727	1730
Carvone	99-49-0	—	—	—	1.30 ± 0.10 ^b^	—	6.45 ± 0.51 ^a^	—	—	—	—	1735	1735
**Esters**													
Octyl acetate	112-14-1	—	—	—	—	—	4.82 ± 0.25 ^a^	—	—	—	—	1482	1481
Ethyl nonanoate	123-29-5	—	—	—	—	0.93 ± 0.26 ^a^	—	—	—	—	—	1537	1537
Linalyl acetate	115-95-7	—	380.94 ± 2.56 ^a^	—	2.35 ± 0.24 ^d^	1.92 ± 0.33 ^d^	8.35 ± 0.59 ^c^	—	37.69 ± 3.79 ^b^	—	—	1557	1555
(−)-Bornyl acetate	76-49-3	3.93 ± 0.17 ^d^	85.76 ± 0.72 ^a^	—	0.60 ± 0.05 ^e^	1.49 ± 0.16 ^d^	5.07 ± 0.28 ^b^	1.33 ± 0.12 ^d^	1.33 ± 0.10 ^d^	0.67 ± 0.03 ^e^	2.51 ± 0.15 ^c^	1578	1584
Terpinene 4-acetate	4821-04-9	2.06 ± 0.16 ^d^	6.73 ± 0.77 ^a^	—	—	—	4.12 ± 0.12 ^b^	—	—	—	1.51 ± 0.15 ^c^	1614	1619
Citronellyl acetate	150-84-5	—	—	—	—	—	4.86 ± 0.45 ^a^	—	—	—	1.61 ± 0.06 ^b^	1660	1657
Geranyl acetate	105-87-3	8.18 ± 1.12 ^d^	171.98 ± 6.28 ^a^	—	—	—	29.49 ± 3.23 ^b^	—	—	—	9.99 ± 0.46 ^c^	1757	1756
Phenethyl acetate	103-45-7	2.70 ± 0.14 ^d^	4.32 ± 0.39 ^a^	2.27 ± 0.16 ^c^	—	1.17 ± 0.23 ^d^	3.53 ± 0.44 ^b^	—	—	—	—	1818	1825
Ethyl cinnamate	103-36-6	—	2.08 ± 0.20 ^b^	—	0.80 ± 0.04 ^c^	—	—	1.17 ± 0.25 ^c^	2.84 ± 0.65 ^a^	—	—	2132	2132
Cinnamyl acetate	103-54-8	4.28 ± 0.42 ^b^	66.01 ± 2.73 ^a^	—	—	—	—	—	—	1.17 ± 0.09 ^b^	—	2154	2156
**Phenols**													
Pinocarveol	6712-79-4	4.20 ± 0.39 ^d^	8.06 ± 0.60 ^b^	—	3.03 ± 0.20 ^c,d^	16.59 ± 2.68 ^a^	2.47 ± 0.25 ^c,d^	7.09 ± 1.28 ^b^	4.49 ± 0.38 ^c^	—	—	1655	1646
Butylated hydroxytoluene	128-37-0	—	—	—	—	—	—	3.21 ± 0.54 ^a^	—	—	—	1916	1919
Methyl eugenol	93-15-2	75.60 ± 9.86 ^a^	2.33 ± 0.12 ^d^	—	2.00 ± 0.19 ^d^	—	14.88 ± 1.44 ^b^	5.78 ± 1.35 ^c^	2.01 ± 0.36 ^d^	0.46 ± 0.05 ^d^	—	2017	2014
4-Methylphenol	106-44-5	2.40 ± 0.25 ^a^	—	—	—	—	—	—	—	—	—	2088	2090
3-Methylphenol	108-39-4	8.39 ± 1.35 ^b^	—	—	—	—	—	—	—	—	—	2096	2091
Eugenol	97-53-0	3.53 ± 0.15 ^c^	3.92 ± 0.38 ^c^	2.62 ± 0.47 ^c^	1.07 ± 0.07 ^c^	172.74 ± 26.91 ^b^	274.62 ± 28.03 ^a^	25.05 ± 2.61 ^c^	1.37 ± 0.19 ^c^	—	0.75 ± 0.15 ^c^	2170	2175
Isoeugenol	97-54-1	—	—	—		1.16 ± 10.15 ^b^	6.19 ± 0.89 ^a^	1.15 ± 0.32 ^b^	—	—	—	2260	2271
2,4-Di-tert-butylphenol	96-76-4	—	—	—	—	—	—	1.20 ± 0.15 ^a^	—	0.52 ± 0.03 ^b^	0.46 ± 0.02 ^b^	2316	2321
**Total**		3014.66 ± 202.47 ^b^	6964.08 ± 176.04 ^a^	1335.85 ± 67.47 ^e^	1126.97 ± 57.43 ^e^	2572.14 ± 262.77 ^c^	2662.65 ± 167.55 ^c^	1759.03 ± 151.78 ^d^	2344.11 ± 125.15 ^c^	367.55 ± 8.55 ^f^	1140.93 ± 33.96 ^e^		

^1^ Not detected or the RI was not found. ^2^ Different subscript letters in the same row for the same item indicate significant differences (*p* < 0.05). ^3^ References KovatsRI values from Flavour.net and the NIST Chemistry WebBook.

**Table 4:** The OAV value of volatile compounds in each group.

No.	Compound	A1	A2	A3	A4	A5	A6	A7	A8	B1	B2	Olfactory Threshold (μg/kg) ^1^
1	N-Pentanal	0.02	0.00	0.00	0.00	0.00	0.00	0.00	0.00	0.00	0.00	22
2	2-Pinene	0.31	3.82	0.14	0.24	0.62	4.61	0.00	0.55	0.63	1.17	6
3	Toluene	0.03	0.09	0.05	0.05	0.08	0.09	0.00	0.07	0.00	0.02	24
4	Dimethyl disulfide	6.57	0.00	8.77	0.00	0.00	0.00	0.00	0.00	0.00	0.00	0.3
5	N-Hexanal	0.94	0.00	0.00	0.30	0.00	0.00	0.00	0.00	0.18	0.18	5
6	β-pinene	0.01	0.27	0.01	0.01	0.02	0.15	0.01	0.03	0.03	0.05	140
7	Sabinen	0.05	0.70	0.00	0.02	0.02	1.22	0.00	0.05	0.02	0.09	37
8	2-Butylfuran	0.00	0.00	0.00	0.00	0.00	0.00	0.00	0.00	0.00	0.21	5
9	3-Carene	0.01	0.11	0.01	0.00	0.03	0.07	0.00	0.01	0.03	0.03	770
10	Diallyl sulfide	0.02	0.00	0.00	0.00	0.00	0.00	0.03	0.04	0.00	0.00	32.5
11	α-Phellandrene	0.07	0.33	0.05	0.02	0.12	0.18	0.05	0.07	0.12	0.30	40
12	β-Myrcene	2.54	57.53	0.00	0.00	7.40	79.08	3.26	6.60	3.01	29.16	1.2
13	α-Terpilene	0.02	0.05	0.01	0.00	0.00	0.07	2.84	0.02	0.04	0.14	85
14	Ethylbenzene	0.00	0.01	0.01	0.01	0.01	0.02	1.95	0.01	0.00	0.00	200
15	Heptanal	0.00	0.00	0.00	0.00	0.00	0.00	0.00	0.00	0.00	0.18	2.8
16	Limonene	0.23	9.24	0.24	0.36	0.68	4.59	0.07	1.08	0.59	2.31	34
17	Eucalyptol	103.70	227.14	47.45	20.84	87.45	123.29	53.04	150.30	26.16	93.71	1.1
18	β-Phellandrene	0.00	0.00	0.00	0.00	0.00	1.76	0.00	0.00	0.00	0.00	36
19	2-Pentylfuran	0.00	0.00	0.00	0.00	0.00	0.00	0.00	0.00	0.14	0.09	6
20	γ-Terpinene	0.05	0.92	0.00	0.09	0.15	1.07	0.04	0.12	0.00	0.30	65
21	Styrene	0.22	1.45	0.80	0.78	1.13	1.35	0.66	1.21	0.61	3.61	3.6
22	O-Cymene	0.52	6.39	0.31	0.41	0.54	5.26	0.71	0.80	0.96	2.64	5
23	Terpinolene	0.01	0.37	0.00	0.00	0.08	0.00	0.03	0.00	0.07	0.23	41
24	Octanal	2.03	5.85	0.00	0.00	3.53	4.46	2.86	4.19	1.48	4.21	0.58
25	2-Heptanol	0.01	0.04	0.00	0.02	0.02	0.00	0.00	0.00	0.00	0.01	65
26	6-Methyl-5-hepten-2-one	0.06	0.07	0.03	0.02	0.08	0.03	0.05	0.07	0.03	0.05	68
27	Dimethyl trisulfide	0.00	0.00	0.00	0.00	0.00	0.00	0.00	95.53	0.00	0.00	0.01
28	2-Nonanone	0.00	0.00	0.00	0.00	0.00	0.00	0.00	0.00	0.00	0.03	40
29	1-Nonanal	3.45	0.00	2.84	7.33	0.00	9.05	0.00	4.19	1.63	2.49	1.1
30	(E)-2-Octenal	0.00	0.00	0.00	0.00	2.60	0.00	0.00	0.00	0.00	0.00	0.34
31	Linalyl oxide	0.01	0.00	0.00	0.00	0.00	0.00	0.00	0.00	0.00	0.00	100
32	Dehydro-P-cymene	0.00	0.00	0.00	0.01	0.03	0.03	0.00	0.00	0.00	0.04	85
33	1-Octen-3-ol	0.00	0.00	0.00	0.58	0.00	0.00	0.74	0.00	0.00	0.00	1.5
34	Octyl acetate	0.00	0.00	0.00	0.00	0.00	0.10	0.00	0.00	0.00	0.00	47
35	Diallyl trisulfide	0.41	3.70	0.72	0.65	0.09	8.40	0.83	2.94	0.00	0.00	30
36	2-Decanone	1.02	3.68	2.40	2.65	3.06	3.53	2.63	3.00	0.00	0.00	3
37	Decanal	0.00	0.00	0.00	5.92	0.00	12.68	9.86	2.16	1.83	1.58	0.5
38	Camphor	0.01	0.43	0.00	0.00	0.01	0.00	0.08	0.01	0.00	0.00	250
39	Benzaldehyde	0.08	0.14	0.02	0.06	0.04	0.00	0.01	0.14	0.12	0.47	300
40	Linalool	202.26	886.80	51.11	163.34	267.01	66.75	270.53	306.29	7.43	32.81	1
41	Linalyl acetate	0.00	0.38	0.00	0.00	0.00	0.01	0.00	0.04	0.00	0.00	1000
42	1-Octanol	0.01	0.00	0.02	0.00	0.00	0.00	0.04	0.00	0.00	0.01	100
43	(−)-Bornyl acetate	0.02	1.14	0.00	0.01	0.02	0.07	0.02	0.02	0.01	0.03	75
44	β-Caryophyllene	1.00	12.54	10.86	2.37	7.16	7.24	1.20	2.71	0.33	0.56	64
45	Terpinen-4-Ol	0.39	0.58	0.35	0.14	0.34	0.56	0.44	0.30	0.05	0.21	340
46	(E)-2-Decenal	0.00	31.41	0.00	3.23	0.00	5.61	4.85	6.70	0.00	4.21	0.3
47	1-Nonanol	0.00	0.00	0.00	0.00	0.00	0.00	0.04	0.00	0.00	0.00	45.5
48	Humulene	0.03	0.45	0.17	0.11	0.28	0.18	0.08	0.12	0.01	0.02	120
49	Estragole	1.87	27.78	0.00	4.86	8.98	6.83	1.07	0.00	0.43	0.37	6
50	(Z)-Citral	0.08	0.64	0.08	0.00	0.00	0.04	0.11	0.28	0.00	0.00	30
51	Guaiene	0.13	0.72	0.00	0.15	0.76	0.00	0.00	0.45	0.10	0.10	20
52	α-Terpineol	0.54	2.95	0.42	0.58	0.98	1.17	1.61	1.12	0.17	0.48	86
53	Borneol	0.09	0.14	0.02	0.00	0.15	0.01	0.10	0.08	0.03	0.06	140
54	1-Dodecanal	0.00	0.00	0.00	0.00	0.00	6.12	0.00	0.00	0.00	0.00	0.5
55	Piperitone	0.00	0.00	0.03	0.00	0.00	0.05	0.00	0.00	0.00	0.03	680
56	(E)-Citral	0.19	0.87	0.19	0.00	0.33	0.00	0.15	0.36	0.04	0.00	32
57	Carvone	0.00	0.00	0.00	0.05	0.00	0.24	0.00	0.00	0.00	0.00	27
58	Geranyl acetate	0.08	4.78	0.00	0.00	0.00	0.82	0.00	0.00	0.00	0.28	36
59	4-Isopropylbenzaldehyde	0.09	0.09	0.05	0.01	0.05	0.07	0.00	0.12	0.04	0.09	60
60	Nerol	0.00	0.07	0.00	0.01	0.02	0.00	0.05	0.02	0.00	0.00	290
61	Phenethyl acetate	0.05	0.22	0.11	0.00	0.06	0.18	0.00	0.00	0.00	0.00	20
62	Anethole	0.17	7.29	0.10	0.24	0.23	0.81	0.26	0.55	0.19	0.47	50
63	Geraniol	5.31	65.49	6.75	10.33	23.52	2.86	33.05	37.12	1.26	7.53	1
64	1-Methylnaphthalene	0.00	0.00	0.00	0.00	0.00	0.00	0.00	0.00	0.01	0.00	75
65	2-Phenylethanol	0.00	0.00	0.00	0.00	0.00	0.00	0.00	0.00	0.02	0.03	60
66	O-Anisaldehyde	0.01	0.00	0.00	0.00	0.00	0.00	0.03	0.01	0.01	0.02	174
67	Caryophyllene oxide	0.00	0.03	0.00	0.00	0.00	0.02	0.00	0.00	0.00	0.00	200
68	Methyl eugenol	0.37	0.03	0.00	0.03	0.00	0.22	0.08	0.03	0.01	0.00	68
69	4-Methoxybenzaldehyde	0.07	0.00	0.00	0.06	0.00	0.00	0.07	0.04	0.02	0.00	27
70	Cinnamaldehyde	0.00	0.17	0.00	0.01	0.02	0.00	0.00	0.10	0.01	0.03	6000
71	4-Methylphenol	0.21	0.00	0.00	0.00	0.00	0.00	0.00	0.00	0.00	0.00	3.9
72	3-Methylphenol	0.19	0.00	0.00	0.00	0.00	0.00	0.00	0.00	0.00	0.00	15
73	Ethyl cinnamate	0.00	0.12	0.00	0.05	0.00	0.00	0.07	0.17	0.00	0.00	17
74	Cinnamyl acetate	0.01	0.44	0.00	0.00	0.00	0.00	0.00	0.00	0.01	0.00	150
75	Eugenol	0.47	1.57	1.05	0.43	69.10	109.85	10.02	0.55	0.00	0.30	2.5
76	Isoeugenol	0.00	0.00	0.00	0.00	0.01	0.06	0.01	0.00	0.00	0.00	100
77	Myristicin	0.35	0.06	0.00	0.05	0.00	0.34	0.08	0.03	0.04	0.00	30
78	Cinnamyl alcohol	0.00	0.03	0.00	0.04	0.00	0.00	0.00	0.02	0.01	0.00	77

^1^ Olfactory threshold data from compilations of odor threshold values in air, water, and other media (Edition 2011). Note: only substances with OAV > 0.1.

**Table 5:** Electronic nose sensor responses to volatile substances formed in different beef bouillons.

Sensor Name	^1^ A1	A2	A3	A4	A5	A6	A7	A8	B1	B2
W1C	0.93 ± 0.00 ^d^	0.92 ± 0.00 ^e^	0.93 ± 0.00 ^d^	0.95 ± 0.00 ^b^	0.93 ± 0.00 ^d^	0.94 ± 0.00 ^b,c^	0.92 ± 0.00 ^e^	0.94 ± 0.00 ^c^	0.95 ± 0.00 ^a^	0.96 ± 0.00 ^a^
W5S	17.64 ± 1.27 ^c,d^	52.42 ± 10.77 ^a^	18.17 ± 1.32 ^c,d^	16.05 ± 0.44 ^d,e^	25.99 ± 1.28 ^b^	19.97 ± 0.61 ^c,d^	16.74 ± 1.76 ^c,d e^	22.19 ± 0.98 ^b,c^	5.55 ± 0.16 ^f^	11.52 ± 1.27 ^e^
W3C	0.95 ± 0.00 ^b^	0.94 ± 0.00 ^c^	0.95 ± 0.00 ^b^	0.96 ± 0.00 ^a^	0.95 ± 0.00 ^b^	0.96 ± 0.00 ^a^	0.94 ± 0.00 ^c^	0.96 ± 0.00 ^a^	0.95 ± 0.00 ^b^	0.96 ± 0.00 ^a^
W6S	1.29 ± 0.01 ^a^	1.32 ± 0.01 ^a^	1.29 ± 0.07 ^a^	1.20 ± 0.00 ^b^	1.23 ± 0.00 ^b^	1.21 ± 0.00 ^b^	1.32 ± 0.04 ^a^	1.19 ± 0.00 ^b^	1.33 ± 0.01 ^a^	1.19 ± 0.00 ^b^
W5C	0.94 ± 0.00 ^c,d^	0.93 ± 0.00 ^e,f^	0.94 ± 0.00 ^c^	0.95 ± 0.00 ^b^	0.94 ± 0.00 ^b^	0.95 ± 0.00 ^b^	0.93 ± 0.01 ^f^	0.95 ± 0.00 ^b^	0.93 ± 0.00 ^d,e^	0.96 ± 0.00 ^a^
W1S	2.00 ± 0.05 ^b^	1.95 ± 0.06 ^b,c^	1.92 ± 0.05 ^c^	1.72 ± 0.01 ^e^	1.85 ± 0.08 ^d^	1.71 ± 0.04 ^e,f^	2.19 ± 0.05 ^a^	1.84 ± 0.02 ^d^	1.65 ± 0.05 ^f^	1.51 ± 0.03 ^g^
W1W	13.66 ± 1.02 ^c^	33.62 ± 4.48 ^a^	14.31 ± 0.91 ^c^	12.88 ± 0.26 ^c^	19.39 ± 1.88 ^b^	16.89 ± 0.47 ^b^	13.09 ± 1.73 ^c^	18.11 ± 0.77 ^b^	3.61 ± 0.16 ^e^	10.27 ± 1.18 ^d^
W2S	2.86 ± 0.09 ^c^	2.94 ± 0.06 ^c^	2.60 ± 0.10 ^d^	2.17 ± 0.02 ^f^	2.37 ± 0.09 ^e^	2.19 ± 0.05 ^f^	3.19 ± 0.09 ^a^	2.29 ± 0.03 ^e^	3.05 ± 0.05 ^b^	1.98 ± 0.04 ^g^
W2W	3.94 ± 0.16 ^e,f^	6.44 ± 0.34 ^a^	3.98 ± 0.17 ^d^	3.67 ± 0.00 ^f^	4.77 ± 0.20 ^b^	4.35 ± 0.10 ^c^	3.89 ± 0.27 ^e,f^	4.27 ± 0.07 ^c^	2.43 ± 0.15 ^f^	3.65 ± 0.23 ^f^
W3S	1.84 ± 0.03 ^b^	1.96 ± 0.03 ^a^	1.76 ± 0.03 ^c^	1.56 ± 0.01 ^e,f^	1.63 ± 0.02 ^d^	1.59 ± 0.01 ^d,e^	1.92 ± 0.05 ^a^	1.56 ± 0.00 ^e,f^	1.93 ± 0.04 ^a^	1.54 ± 0.01 ^g^

^1^ Different subscript letters in the same row for the same item indicate significant differences (*p* < 0.05).

**Table 6:** Sensory analysis of 10 groups of beef bouillons.

Attributes	^1^ A1	A2	A3	A4	A5	A6	A7	A8	B1	B2
Overall acceptability	8.88 ± 0.74 ^a^	6.18 ± 0.55 ^f^	7.02 ± 0.71 ^e^	6.14 ± 0.72 ^f^	7.18 ± 0.62 ^e^	7.58 ± 0.67 ^d^	8.46 ± 0.85 ^b^	7.78 ± 0.70 ^c,d^	6.22 ± 0.54 ^d^	7.96 ± 0.77 ^c^
Beef like	3.60 ± 0.80 ^b,c^	4.70 ± 0.90 ^a^	3.60 ± 0.66 ^b,c^	2.60 ± 0.66 ^d^	4.00 ± 1.00 ^a,b,c^	4.30 ± 1.00 ^a,b^	3.70 ± 0.78 ^b,c^	3.40 ± 0.80 ^c^	2.60 ± 0.49 ^d^	3.70 ± 0.64 ^b,c^
Fat like	2.20 ± 0.60 ^a^	1.40 ± 0.49 ^b^	2.30 ± 0.46 ^a^	1.60 ± 0.49 ^b^	1.40 ± 0.49 ^b^	1.30 ± 0.46 ^b^	1.50 ± 0.50 ^b^	1.30 ± 0.46 ^b^	1.30 ± 0.46 ^b^	1.70 ± 0.46 ^b^
Spice like	6.90 ± 0.70 ^c,d^	9.20 ± 0.75 ^a^	5.30 ± 0.64 ^e^	6.30 ± 0.64 ^d^	7.10 ± 0.54 ^c^	5.50 ± 0.67 ^e^	7.00 ± 0.63 ^c^	8.00 ± 0.63 ^b^	5.30 ± 0.78 ^e^	7.10 ± 0.70 ^c^
Salty taste	5.60 ± 0.80 ^c,d^	6.30 ± 0.64 ^a,b^	5.80 ± 0.60 ^b,c^	6.60 ± 0.49 ^a^	4.90 ± 0.70 ^e^	5.00 ± 0.63 ^d,e^	6.10 ± 0.70 ^a,b,c^	6.10 ± 0.70 ^a,b,c^	4.00 ± 0.77 ^f^	6.70 ± 0.46 ^a^
Astringency taste	2.30 ± 0.46 ^a,b^	2.00 ± 0.63 ^a,b,c^	2.40 ± 0.49 ^a,b^	1.90 ± 0.54 ^a,b,c,d^	1.50 ± 0.50 ^c,d^	2.20 ± 0.60 ^a,b^	2.00 ± 0.45 ^a,b,c^	1.80 ± 0.60 ^b,c,d^	1.40 ± 0.49 ^d^	1.40 ± 0.49 ^d^
Sour taste	1.70 ± 0.64 ^a^	1.20 ± 0.40 ^b,c^	1.50 ± 0.50 ^a,b,c^	1.60 ± 0.49 ^a,b^	1.90 ± 0.30 ^a^	1.80 ± 0.60 ^a^	1.50 ± 0.50 ^a,b,c^	1.50 ± 0.50 ^a,b,c^	1.80 ± 0.40 ^a^	1.10 ± 0.30 ^c^
Bitter taste	2.20 ± 0.40 ^d^	3.10 ± 0.70 ^a,b,c^	2.70 ± 0.46 ^b,c,d^	3.60 ± 0.66 ^a^	2.50 ± 0.50 ^d^	3.20 ± 0.40 ^b,c^	2.60 ± 0.49 ^c,d^	2.50 ± 0.50 ^d^	2.40 ± 0.49 ^d^	2.30 ± 0.46 ^d^
Umami taste	7.90 ± 0.83 ^a^	5.90 ± 0.70 ^e^	6.00 ± 0.63 ^d,e^	6.30 ± 0.64 ^d,e^	6.70 ± 0.78 ^d,e^	7.10 ± 0.70 ^b,c^	7.80 ± 0.75 ^a,b^	7.10 ± 0.70 ^b,c^	6.10 ± 0.70 ^d,e^	7.20 ± 0.75 ^a,b,c^

^1^ Different subscript letters in the same row for the same item indicate significant differences (*p* < 0.05).

## Data Availability

Not applicable.
